# Supplementary material for: Seminoma and Embryonal Carcinoma Footprints Identified by Analysis of Integrated Genome-Wide Epigenetic and Expression Profiles of Germ Cell Cancer Cell Lines
Source: PLoS One. 2014 Jun 2;9(6):e98330. doi: 10.1371/journal.pone.0098330 (PMC4041891; doi:10.1371/journal.pone.0098330)
Supplement: File S1 — ZIP file containing DMRforPairs output for significant regions. Please start from the html files. (ZIP) [file pone.0098330.s008.zip › figures/36356.pdf]

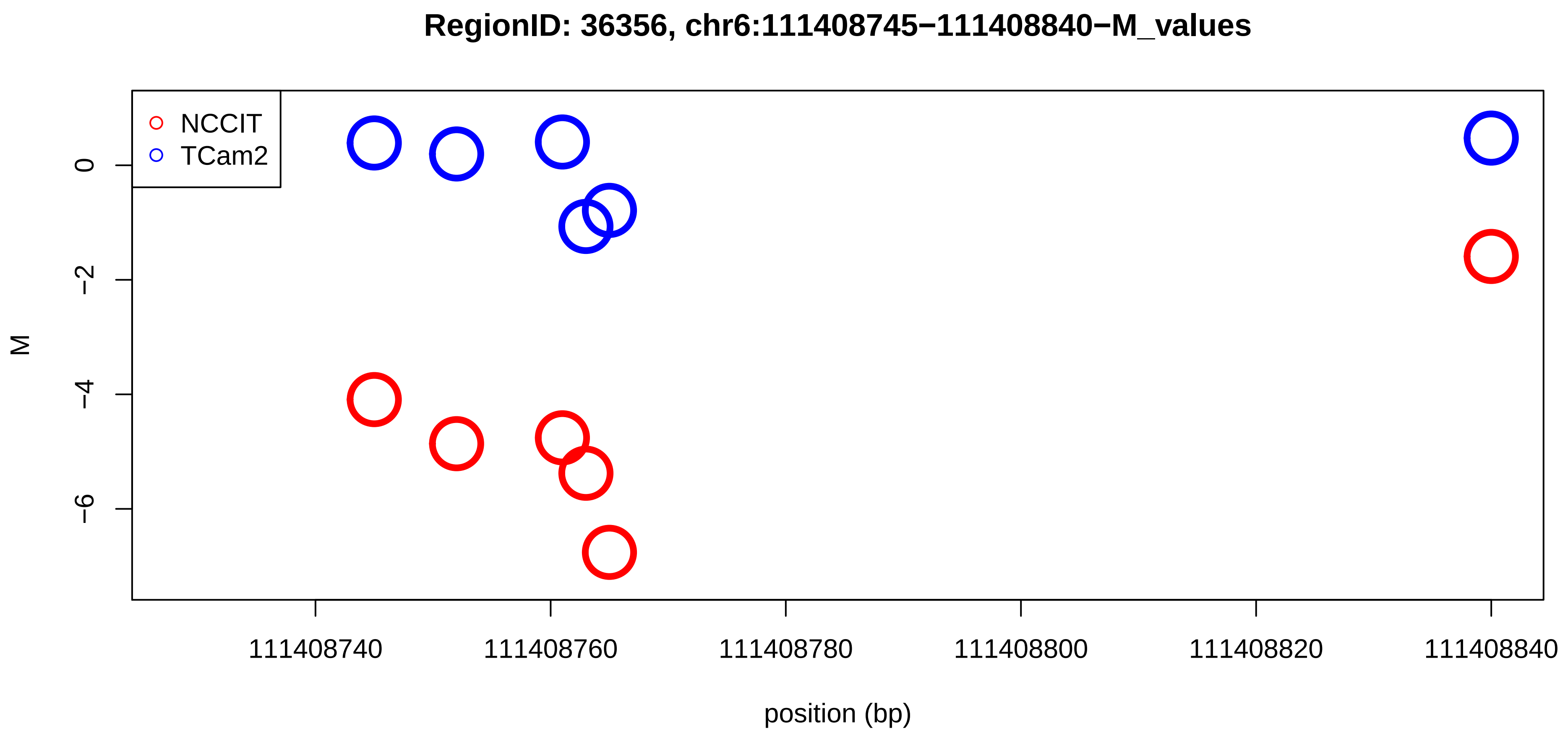

RegionID: 36356, chr6:111408745−111408840−M_values
NCCIT
TCam2
0
−2
−4
−6
M
111408740
111408760
111408780
111408800
111408820
111408840
position (bp)

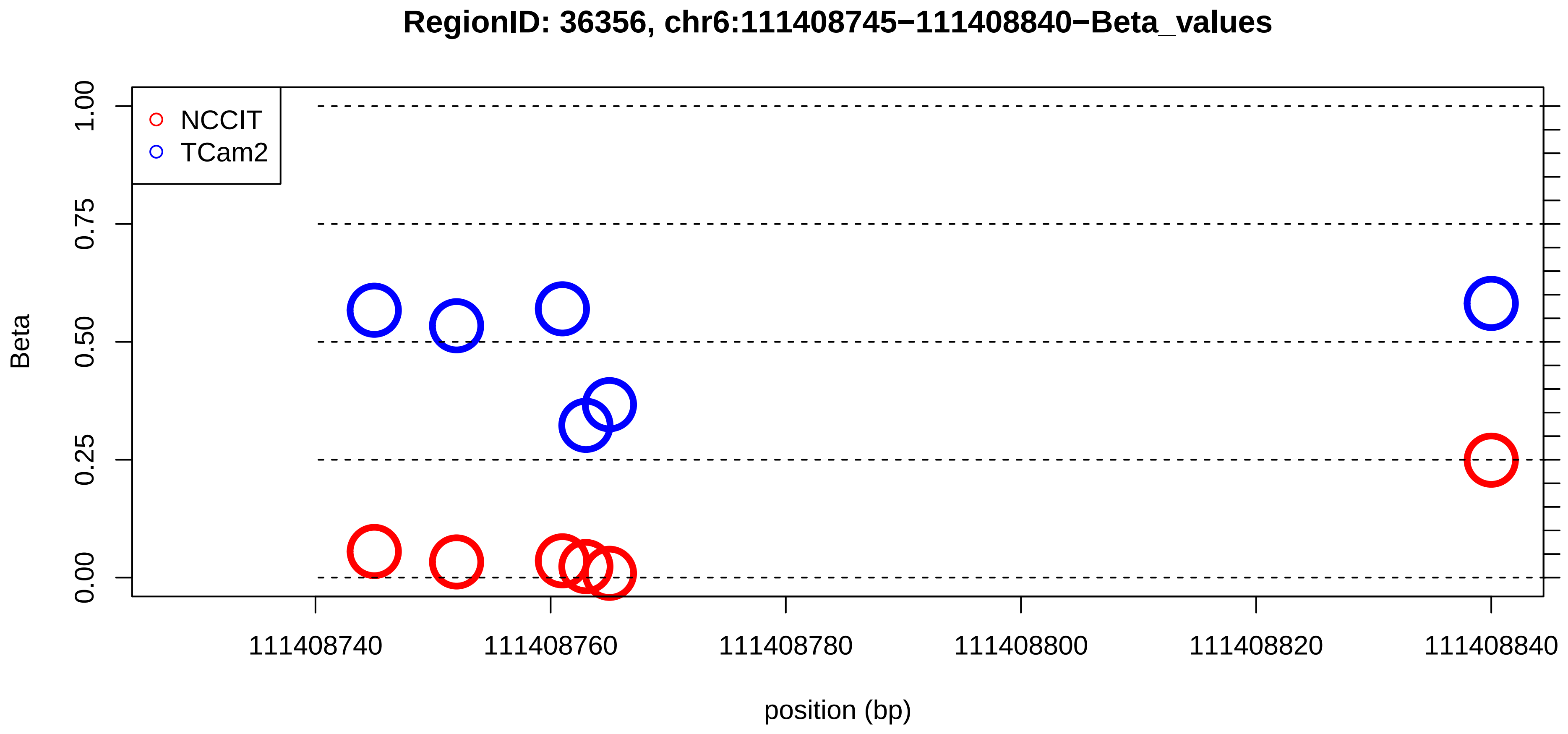

RegionID: 36356, chr6:111408745−111408840−Beta_values
NCCIT
TCam2
1.00
0.75
0.50
0.25
0.00
Beta
111408740
111408760
111408780
111408800
111408820
111408840
position (bp)
